# Supplementary material for: Eukaryotic CRFK Cells Motion Characterized with Atomic Force Microscopy
Source: Int J Mol Sci. 2022 Nov 19;23(22):14369. doi: 10.3390/ijms232214369 (PMC9692694; doi:10.3390/ijms232214369)
Supplement: Supplementary file 1 [file ijms-23-14369-s001.zip › Supplementary Material.pdf]

Eukaryotic CRFK cells motion characterized with atomic force microscopy

Maria Zamora-Ceballos [1], Juan Barcena [2] and Johann Mertens [1]

[1] Madrid Institute for Advanced Studies in Nanoscience (IMDEA Nanoscience), Campus de Cantoblanco, 28049 Madrid, Spain

[2] Centro de Investigación en Sanidad Animal (CISA-INIA/CSIC), Valdeolmos, 28130 Madrid, Spain.

Table S1

cell	lenght	width	type of movement	distance	speed
	(nm)	(nm)		(µm)	(nm/min)
1	81	23	changing direction	8	57
2	>98	16	linear	14	96
3	80	18	linear	17	115
4	66	21	changing direction	7	48
5	51	20	linear	48	324
6	>60	28	linear	23	154
7	34	28	changing direction	23	243
8	41	23	stop	8	54
9	48	28	linear	28	189
10	98	14	linear	65	439
11	67	19	stop	14	95
12	49	22	stop/changing direction	10	70
13	47	22	stop	9	61
14	61	16	linear	14	92
15	63	25	stop	7	50
16	34	36	changing direction	14	93
17	81	20	linear	45	309
18	91	29	linear	17	116
19	104	23	linear	16	112
20	54	33	stop	8	56
21	106	19	linear (up and down)	43	29
22	94	13	linear (up and down)	15	101
23	44	38	stop	11	72
24	84	31	changing direction	14	97
25	84	22	linear (up and down)	24	163
26	>36	14	linear	15	102
27	64	47	linear	24	166
28	67	33	changing direction	17	114
29	>49	40	changing direction	37	253
30	67	32	linear	12	85
31	62	31	stop	8	53

Table S1: Optical microscope characterization of the motion of CRFK cell onto glass substrates shows the dynamics and morphologies of the cells as well as the nature of their motion during displacement.

Figure S1

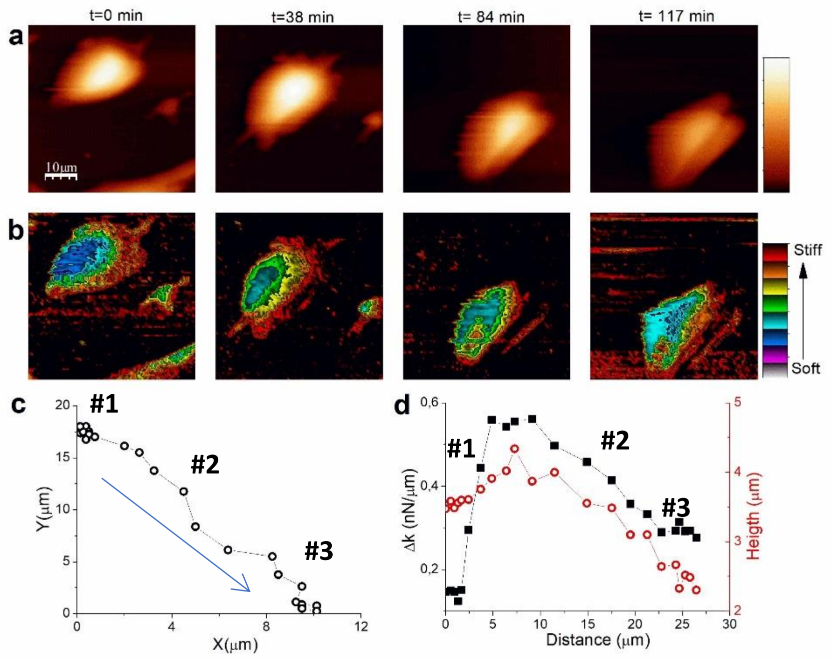

Figure S1. Series of **a)** topographic (Z-scale = 4 µm) and **b)** Spring constant k (Z-scale: 2 nN/µm), images (50 µm × 50 µm) recorded in real time for a single CRFK cell during its motion on glass surface for t = 0, 38, 84 and 117 min. respectively. **c**) Trajectory of the cell plotted as the position of the cell for the aligned images. **d)** Plot of $\Delta k_{rear\text{-}front}$ (black) and cell maximum height (red), as function of the cell's traveled distance.

Time-lapse AFM images were recorded in cells culture medium (DMEM) at 37 ºC. 22 successive images were collected at fixed time intervals of ≈ 6 minutes. We monitored the topography (**Figure S1a**) and spring constant (**Figure S1b**) response of the cell during its motion at glass slide surface. During the first 30 minutes, the cell is almost stopped (**Figure S1c**). This phase 1 (#1) is characterized by constant Δk≈ 0.1 nN/µm and height ≈ 3.5 µm (**Figure S1d**). The cells start moving when Δk increase up to ≈ 0.55 nN/µm and cell height reaches ≈ 5 µm. During this linear motion (#2), the cell travels ≈ 10 µm, Δk and cell height decrease linearly to ≈ 0.3 nN/µm and ≈ 3.5 µm respectively until the cell stops again in a phase 3 (#3) where Δk and the cell height keep unchanged again.

Figure S2

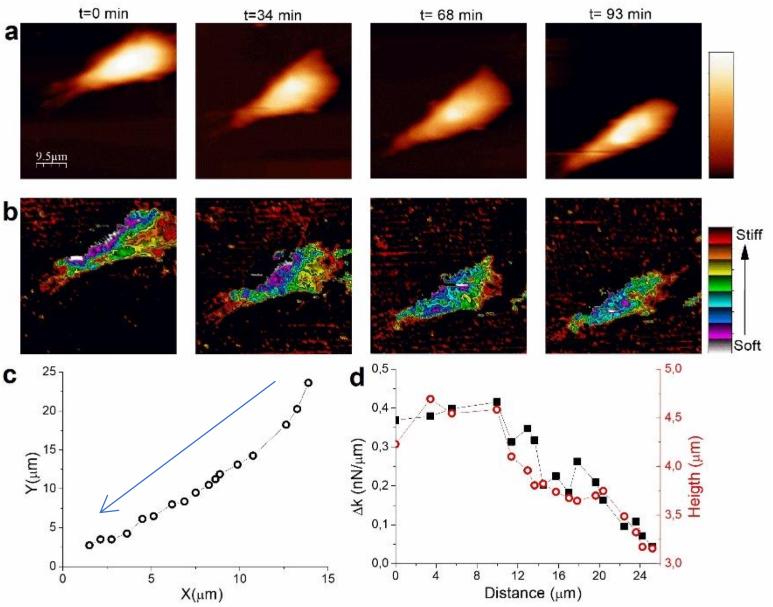

Figure S2. Series of **a)** topographic (Z-scale = 4 µm) and **b)** Spring constant k (Z-scale: 3.6 nN/µm), images (50 µm × 50 µm) recorded in real time for a single CRFK cell during its motion on glass surface for t = 0, 28, 57 and 86 min. respectively. **c**) Trajectory of the cell plotted as the position of the cell for the aligned images. **d)** Plot of $\Delta k_{rear\text{-}front}$ (black) and cell maximum height (red), as function of the cell's traveled distance.

Time-lapse AFM images were recorded in cells culture medium (DMEM) at 37 ºC. 17 Successive images were collected at fixed time intervals of ≈ 6 minutes. We monitored the topography (**Figure S2a)** and spring constant **(Figure S2b)** response of the cell during its motion at glass slide surface. The cell travels 25 µm mainly from the upper right to the center of the image (**Figure S2c, blue arrow**). During the linear motion, Δk and cell height decrease linearly from ≈ 0.4 to ≈ 0.05 nN/µm and from≈ 4.5 to 3.2 µm respectively (**Figure S2d**). Surprisingly the cell does not move along its large axis and it rotates slightly counterclockwise while moving.

Figure S3

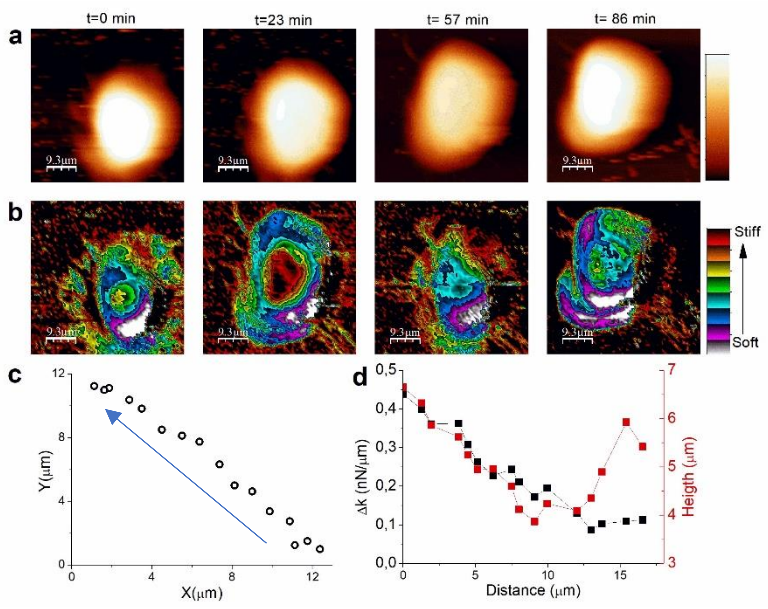

Figure S3. Series of **a)** topographic (Z-scale = 5 µm) and **b)** Spring constant k (Z-scale: 1.2 nN/µm), images (50 µm × 50 µm) recorded in real time for a single CRFK cell during its motion on glass surface for t = 0, 23, 57 and 86 min. respectively. **c**) Trajectory of the cell plotted as the position of the cell for the aligned images. **d)** Plot of $\Delta k_{rear\text{-}front}$ (black) and cell maximum height (red), as function of the cell's traveled distance.

Time-lapse AFM images were recorded in cells culture medium (DMEM) at 37 ºC. 16 successive images were collected at fixed time intervals of ≈ 6 minutes. We monitored the topography (**Figure S3a)** and spring constant **(Figure S3b)** response of the cell during its motion at glass slide surface. The cell shows a linear change in position traveling 17 µm from the bottom left to the upper right of the image (**Figure S3c, blue arrow**). During the linear motion, Δk and cell height decrease linearly from ≈ 0.45 to ≈ 0.1 nN/µm and from≈ 6.5 to 4 µm respectively (**Figure S3d**) until the cell nearly stop. Then the cell height increases again to ≈ 6 µm during the last 20 minutes of the experiment.

Figure S4

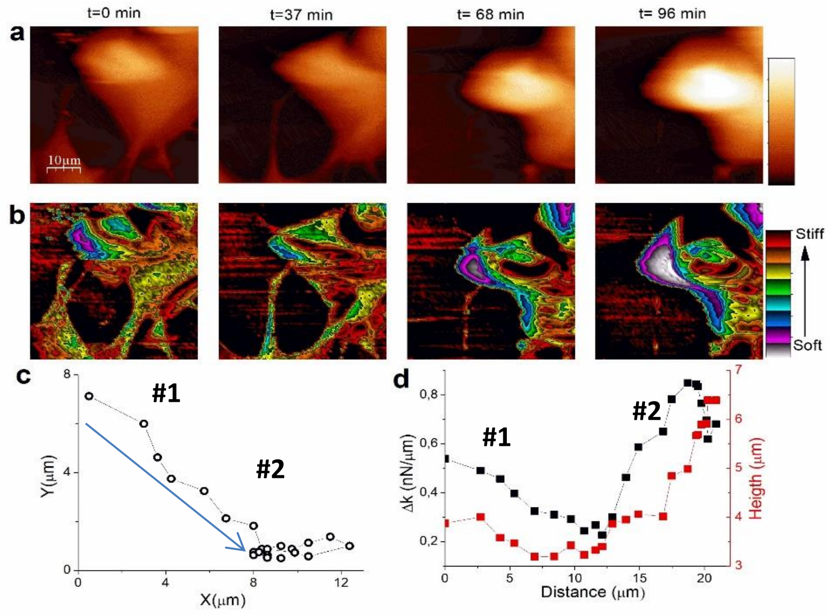

Figure S4. Series of **a)** topographic (Z-scale = 6 µm) and **b)** Spring constant k (Z-scale: 1.5 nN/µm), images (50 µm × 50 µm) recorded in real time for a single CRFK cell during its motion on glass surface for t = 0, 37, 68 and 96 min. respectively. **c**) Trajectory of the cell plotted as the position of the cell for the aligned images. **d)** Plot of $\Delta k_{rear\text{-}front}$ (black) and cell maximum height (red), as function of the cell's traveled distance.

Time-lapse AFM images were recorded in cells culture medium (DMEM) at 37 ºC. 22 successive images were collected at fixed time intervals of ≈ 6 minutes. We monitored the topography (**Figure S4a)** and spring constant **(Figure S4b)** response of the cell during its motion at glass slide surface. This cell is strongly connected to it neighboring cells from the upper left and the bottom of the image. During the first 50 minutes (blue arrow), the cell linearly travels 8 µm (**Figure S4c, #1**). Δk and cell height decrease linearly from ≈ 0.55 to 0.2 nN/µm and from≈ 4 to 3 µm respectively (**Figure S4d**). The second phase (#2) is more complex as the cell changes its direction. The cell is still moving but it also rotates counter clockwise to orientate following the X-axis then nearly stops. While rotating, Δk and cell height increases from ≈ 0.2 to 0.7 nN/µm and from≈ 3 to 6.5 µm respectively.

Figure S5

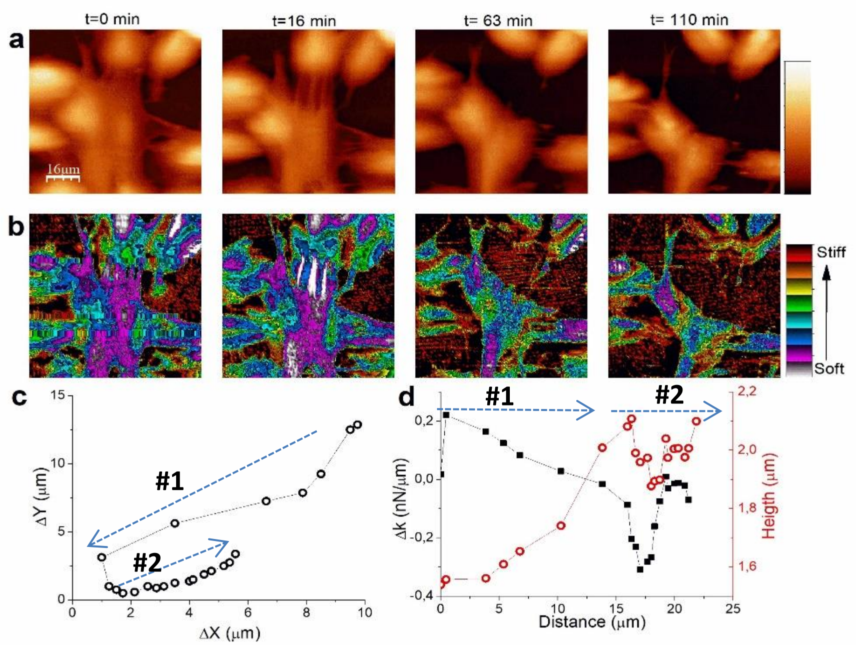

Figure S5. Series of **a)** topographic (Z-scale = 4 µm) and **b)** Spring constant k (Z-scale: 2 nN/µm), images (80 µm ×80 µm) recorded in real time for a single CRFK cell during its motion on glass surface for t = 0, 16, 63 and 110 min. respectively. **c**) Trajectory of the cell plotted as the position of the cell for the aligned images. **d)** Plot of $\Delta k_{rear\text{-}front}$ (black) and cell maximum height (red), as function of the cell's traveled distance.

Time-lapse AFM images were recorded in cells culture medium (DMEM) at 37 ºC. 22 successive images were collected at fixed time intervals of ≈ 6 minutes. We monitored the topography (**Figure S5a)** and spring constant **(Figure S5b)** response of the cell during its motion at glass slide surface. We characterized the motion central cell (dotted blue circle), because it showed the largest changes in position during the measurements. The cell starts moving downward about 10 µm and left (about 9 µm), during 40 min (**Figure S5c, #1**). This movement is characterized by a decrease in the k cell junctions to the upper cell (**Figure S5b, Image 2, red arrow**). Then it stops and changes direction to move slower in the opposite direction during the rest of the experiment (# 2). During the phase 1 (#1), Δk (**Figure S5d, black curve)** is positive to ≈ 0.2 nN/µm and decreases linearly until the cell stops when Δk = 0 nN/µm. Cell height (**Figure S5d, red curve**) increases from ≈ 1.6 to 2.1 µm because the cell detaches from the upper cell when softer junctions break, leading to a change in cell spreading during motion. Then k reverses and becomes negative to Δk = -0.35 nN/µm during phase 2 (#2) as the cell changes its trajectory to move to the opposite direction. Cell height decreases when k dissymmetry increases emphasizing the link between cytoskeleton reorganization and cell morphology.

Description of the additional Supplementary Movie.

226 min JM-AFM of 40 images sequence of the CRFK cell motion after images correlation to correct for drift. Movie parameters: frame size: 73 µm. Full color Z-scale: 3 µm. Image acquisition speed: 10 Hz.
